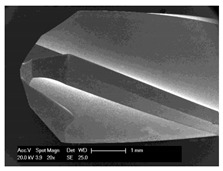# The Influence of Thrust Force on the Vitality of Bone Chips Harvested for Autologous Augmentation during Dental Implantation

**DOI:** 10.3390/ma12223695

**Published:** 2019-11-09

**Authors:** Anas Ben Achour, Carola Petto, Heike Meißner, Dominik Hipp, Andreas Nestler, Günter Lauer, Uwe Teicher

**Affiliations:** 1 Institute of Manufacturing Technology, Technische Universität Dresden, 01062 Dresden, Germany; andreas.nestler@tu-dresden.de; 2 Department of Oral and Maxillofacial Surgery, University Hospital Carl Gustav Carus Dresden, Technische Universität Dresden, Fetscherstraße 74, 01307 Dresden, Germany; Carola.Petto@uniklinikum-dresden.de (C.P.); guenter.lauer@uniklinikum-dresden.de (G.L.); 3 Department of Prosthetic Dentistry, University Hospital Carl Gustav Carus Dresden, Technische Universität Dresden, Fetscherstraße 74, 01307 Dresden, Germany; heike.meissner@uniklinikum-dresden.de; 4 Fraunhofer Institute for Material and Beam Technology IWS, Winterbergstraße 28, 01277 Dresden, Germany; dominik.hipp@iws.fraunhofer.de; 5 Fraunhofer Institute for Machine Tools and Forming Technology IWU, Nöthnitzer Straße 44, 01187 Dresden, Germany; uwe.teicher@iwu.fraunhofer.de

**Keywords:** drilling, thrust force, bone, augmentation, bone chips, vitality, implantation

## Abstract

Bone drill chips that are collected during implant site preparation can be reused as autologous bone-grafting material for alveolar ridge augmentation. This study characterized five market-leading implant drill sets regarding their geometric properties and ability to produce vital bone chips. The drill geometry of each tool of five commercial implant drill sets was characterized while using optical profile projector devices and SEM. Bone chips were collected during the in vitro preparation of porcine jaw bone with the various drill sets. Produced bone chip masses were measured. The bone chips were cultured in vitro and the number of outgrown cells was determined and measurand for vitality. Furthermore, the thrust force and cutting torque were recorded to examine the mechanical loads of the manual drilling process. The tool geometry and set configuration of one out of five implant drill sets appears to be superior regarding chip mass, vitality, and thrust force. It could be proven that there is a correlation between vitality and thrust force. The thrust force is influenced by the cutting behavior of the tool, which in turn depends on the geometry of the tool. The tool geometry has an influence on the vitality of the augmentation material due to this relationship.

## 1. Introduction

A sufficient bone quality and quantity of the alveolar ridge is essential for the successful integration of a dental implant [1]. In the case of deficient alveolar ridges, bone volume can be increased by an augmentation with autologous bone rings [2] or autologous bone grafts, because the use of body´s own tissue is the safest and most compatible method for the patient [3,4]. For larger defects, whole bone blocks are transplanted, which have to be removed from the patient elsewhere [5]. Compact bone blocks can be obtained from the iliac crest or the tibia head, with or without cancellous portions, while using trephines. The disadvantage of this method is the traumatizing temperature development during drilling and the required coolant fluid, which affects the material that is to be extracted. Furthermore, the extracted material must be crushed with the help of bone mills prior to use.

For smaller defects, particulate bone can be used [6,7]. A relatively simple method to obtain autologous particulate bone-grafting material is the removal from the retromolar region or the chin region by using bone scrapers or raspatories. The major disadvantage of this method is the fact that these bone chips that ablated from the surface of the bone consist of cortical cells that have lower regenerative potential than cells that reside in underlying cancellous bone [5]. Anyway, it is proven that these manual or piezoelectric instruments can be safely used for sinus floor augmentation [8].

Another disadvantage of these methods for harvesting autologous bone-grafting material from the retromolar region, chin, iliac crest, or the tibia head is that they generally require an additional surgery at a separate wound side. Therefore, the patient is exposed to further discomforts and risks before the tooth implantation surgical procedures. It is possible to collect the bone chips produced while implant site drilling, which normally would be discarded and reuse it as autologous bone-grafting material, to avoid these additional burdens [7,9].

These bone chips, which are a byproduct of the preparation of the implant site, consist of a mixture of cancellous and cortical bone chips. While the cortical bone particles have high mechanical stability, the cancellous bone chips comprise a larger number of cells. Cancellous bone contains different cell types, particularly osteocytes, osteoclasts, osteoblasts, and stromal cells, and all of these cells have osteogenic, osteoconductive, and osteoinductive effects, as well as the inorganic components of the bone [10]. Therefore, it is important not to disrupt or irreversibly damage these cells, as well as preserve their vitality and thus their regenerative potential, because this has a significant influence on the success of augmentation.

The same factors that have an influence on the surrounding bone during the preparation of the implant site and, in consequence, on the subsequent osseointegration of the implant also affect the bone chips extracted. The multifactorial process of energy input during machining of bones is very complex and the clinician cannot directly assess it. With a given instrument geometry, the input of mechanical energy into the bone tissue depends on the rotational speed and feed rate, and it is converted into cutting energy and frictional heat during drilling [11].

The work that is performed during the surgery is usually evidence-based; the speed setting is carried out according to the recommendations of the instrument manufacturers and can be regarded as constant. Thus, elevated temperatures cause osteonecrosis [12] and the disruption of cells reduces their number as well. On the one hand, heat generation can be reduced by irrigation [13]. Although irrigation is an effective method to prevent heat damage, it has some disadvantages in terms of the use of drill chips as augmentation material. There is evidence that irrigation reduces the regenerative potential of bone chips and increases the risk of bacterial contamination [14,15]. On the other hand, the properties of the drill and the process parameters also play a crucial role. The friction heat is reduced at the origin by drilling at lower speeds in order to avoid these risks [16]. This method can also reduce the mechanical stress on the cells and prevent disruption.

It is more difficult to assess the effects of a varying thrust force. In the technical field of machining, the feed per tooth *f_z_* in mm is set on the machine, depending on the material to be machined and it is usually carried out under absolutely standardized conditions (workpiece with defined geometry, defined hardness, homogeneous material). In the case of hand-guided drilling of the implant cavity, the control of the machining process and, in particular, the thrust force, is very individual and it is influenced by the feedback that the dentist receives from possibly inhomogeneous patient jaws of unknown bone density.

Not only the drilling speed, but also other process parameters, affect the heat generation and mechanical stress: drilling feed rate, applied axial load, drilling depth, use of graduated versus one-step drilling, intermittent versus continuous drilling. However, the properties of the drill itself are likely to also have a great impact in terms of temperature and mechanical load [17]. This includes drill geometry (shape, diameter, point angle), sharpness, drill material, and wearing [11]. The drill geometry seems to influence particle size and the shape of the bone chips [18]. If bone particles are too small or shaped unfavourably, the included cells are disrupted and thus the regenerative potential of the cells is lost [19,20,21].

**Figure 1:**
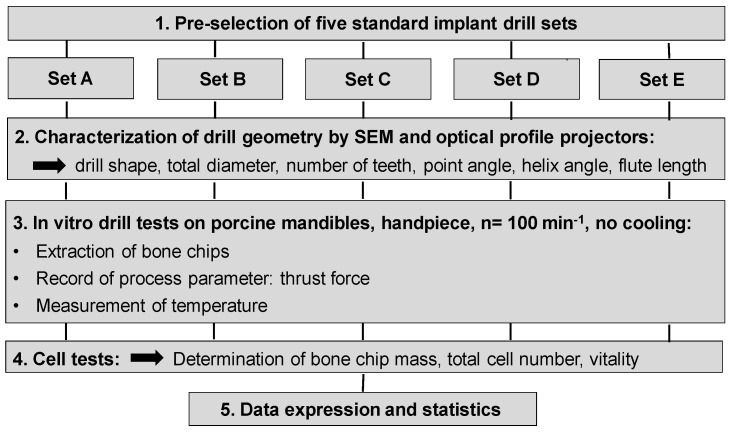
Schematic diagram of the study design.

This raises the question of the optimal drill design, which limits both heat development and mechanical stress in such a way that the vitality of the cells that are contained in the bone chips is optimally preserved. Currently, there is a multitude of implant drills and systems available to meet the individual conditions and needs of patients. This led to the objective of this study to characterize the market-leading drill sets with regard to their geometric properties, their ability to produce vital chips, and to analyse the influence of the thrust force in the manual drilling process, which has not been investigated in detail so far. Indicators for the comparative assessment of the tool geometries with regard to their suitability for optimum chip generation were chip vitality and chip mass generated by the various drill sets, as well as the thrust force process parameters that were recorded during the drilling process. An adapted study design was developed for this purpose (Figure 1).

## 2. Materials and Methods

### 2.1. Implant Drills

**Figure 2:**
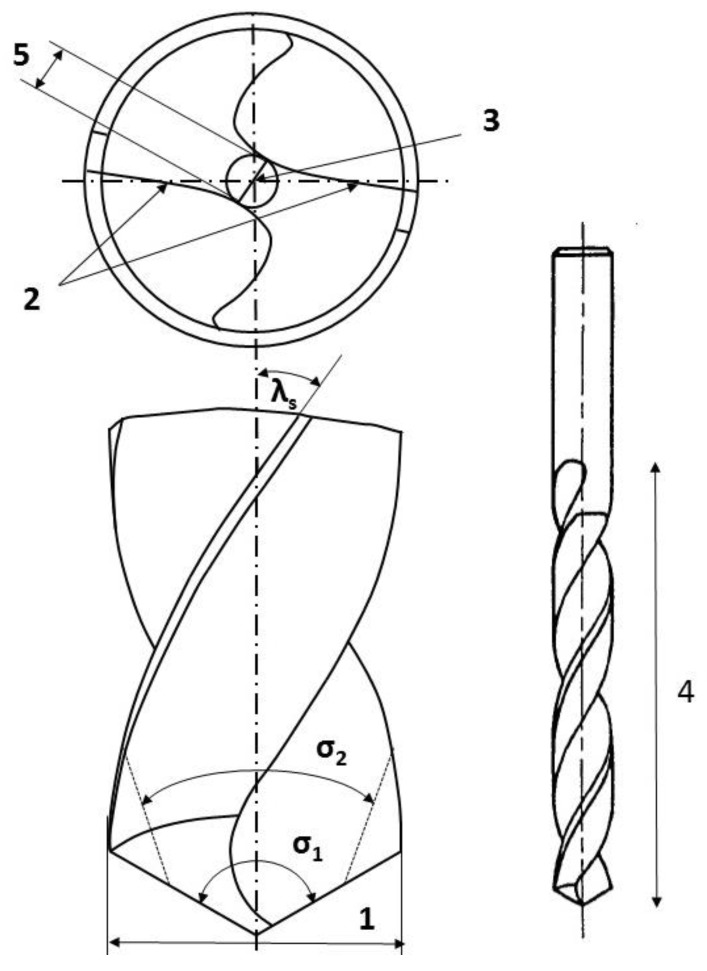
Drill geometry with: (1) tool diameter, (2) main cutting edges, (3) chisel edge, (4) flute length, (5) chisel edge length/core thickness, (λ_s_) helix angle, (σ_1_) point angle, and (σ_2_) point angle for segmented part.

Drilling tools of the market-leading suppliers of implant systems (CAMLOG Group, Basel, Switzerland; Dentsply International Inc. (XIVE), Charlotte, USA; Nobel Biocare AG, Kloten, Switzerland; SIC invent AG, Basel, Switzerland; Straumann GmbH, Berlin, Germany) were analysed and their tool geometry parameters determined as the starting point of the investigations in order to understand the mechanisms of drill chip formation and temperature development better. The following parameters were evaluated for the characterization of the drill geometry (Figure 2): drill shape, tool diameter, number of teeth, flute length, point angle, and helix angle.

For this purpose, each tool was analysed with optical profile projector devices (Table 1) and scanning electron microscope XL 30 ESEM (Philips, Eindhoven, The Netherlands). 

### 2.2. Bone Material

In total, 75 fresh porcine mandibles were obtained from a slaughterhouse (Biofleisch- und Wurstwaren Vera und Bernhard Probst GbR, Dresden, Germany) and transported in phosphate-buffered saline solution (PBS). The mandibles were tested within 4 h post mortem. The alveolar ridge contained individually different proportions of cancellous and cortical bone. To work standardized, we decided to examine a region where cancellous bone could be extracted without cortical bone. For this purpose, the cartilage tissue was removed from the head of the temporomandibular joint (TMJ) down to the mineralized cancellous bone while using a scalpel, and thus preventing chondrocytes from contaminating the cell culture. The drilling holes were inserted into these prepared TMJ heads. Each TMJ was drilled by only one implant drill set until the final diameter of the set.

### 2.3. Conducting the Extraction of Chips

**Figure 3:**
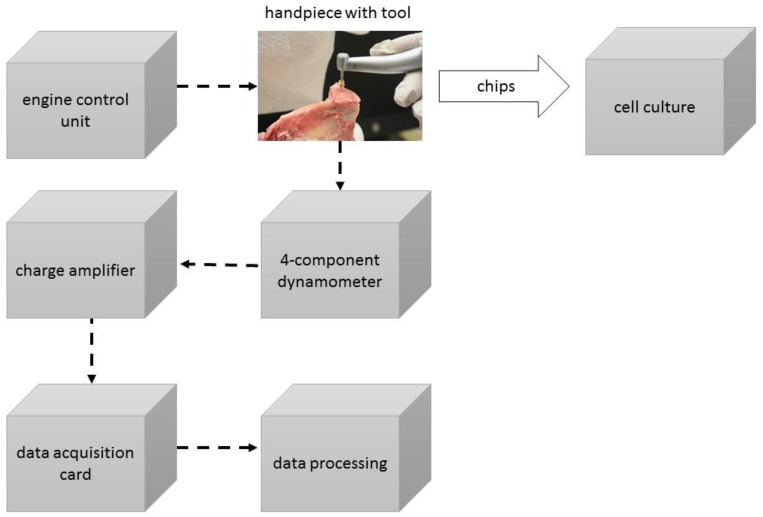
Schematic diagram of the experimental set-up.

After examination of the five different implant drill sets, drilling tests were performed with an Elcomed 200 system (W&H Dentalwerk Bürmoos GmbH, Bürmoos, Austria) and a surgical contra-angled handpiece with a gear ratio of 10:1. The drilling speed was consistently 100 rpm. The depth of the drilling hole was determined with 10 mm. Mandibles were fixed into a vice attached to a piezo-electric Kistler dynamometer 9272 (Kistler Instrumente AG, Winterthur, Switzerland) to measure the thrust force (Figure 3). The signals of the dynamometer were amplified by a charge amplifier 5007 (Kistler Instrumente AG, Winterthur, Switzerland) and digitized with a data acquisition card Goldammer USB basic (Soft- & Hardware Entwicklung Goldammer GmbH, Wolfsburg, Germany). DIAdem 8.1 was the software to analyze the signals (National Instruments, Austin, TX, USA). This procedure is already described in detail regarding the evaluation of the thrust force during low speed drilling [22]. 

### 2.4. Temperature Measurement

Random tests were carried out with the aid of a thermographic measuring method in order to estimate whether effects from heat generation during mechanical energy input by drilling with the given parameters induce temperatures in the cell-damaging range above 40 °C.

Thermal analysis was undertaken with an uncooled microbolometer infrared focal plane array that was integrated in an infrared camera Infratec PIR uc 180 (Infratec GmbH, Dresden, Germany). Evaluation was done with the software IRBIS 3 (Infratec GmbH, Dresden, Germany). The setting of the emissivity ε was done with water after two-point calibration of a slightly wet drill tip with 0.56.

The maximum temperatures were measured after the drilling process at the tool tip, immediately after the drill was retracted from the drilling hole.

### 2.5. Analysis of the Bone Chips

#### 2.5.1. Determination of the Chip Mass

The theoretical determination of the resulting chip mass is based on the measurement of the tool diameters while considering the depth of the drilling hole and a tissue density of cancellous bone. An ideal cylinder was assumed as the basis for the calculation to simplify the volume calculation. The theoretical drilling area *A_theor_* of the drilling hole was calculated according to Equations (1) and (2), respectively:

(1) $$A_{theor}=\;\frac{\pi }{4}\cdot d_{tool}{}^{2}$$

or in general for a circular ring area for drill out cycles

(2) $$A_{theor}=\;\frac{\pi }{4}\cdot \left(d_{tool\left(n\right)}{}^{2}-d_{tool\left(n-1\right)}{}^{2}\right)$$

With the theoretical drilling area and the drilling depth *l*, the theoretical cylinder volume was the following:

(3) $$V_{theor}=A_{theor}\cdot l$$

The theoretical cylinder volume (Equation (3)) had to be multiplied with the density of the bone to calculate the theoretical bone chip mass (Equation (4)). For this calculation, the measured tissue density of cancellous bone from Giesen et al. (2001) with $\rho_{bone}$ = 2.1 g/cm^3^ was used [23].

(4) $$m_{theor}=V_{theor}\cdot \rho_{bone}$$

The practical determination of the chip mass of the bone chips that were produced during the drilling process was performed by determining the weight using the Sartorius Basic BA 100S-OD1 electronic balance (Sartorius, Goettingen, Germany) with a linearity deviation of ±0.2 mg.

#### 2.5.2. Determination of the Chip Sizes

Scanning electron microscopic examinations for the evaluation of the drill chip preparations and their measurement were carried out with the scanning electron microscope XL 30 ESEM (Philips, Eindhoven, The Netherlands) in wet room mode and in conventional mode. The samples were sputtered with gold while using the Cressington sputtercoater 108 (Cressington Scientific Instruments UK, Watford, UK).

#### 2.5.3. Cell Culture

Bone chips were collected in PBS supplemented with 100 µg/mL gentamicin, 200 U/mL penicillin, 200 µg/mL streptomycin and 5 µg/mL amphotericin B (all from Sigma-Aldrich Chemie GmbH, Taufkirchen, Germany). After weight determination, the bone chips were transferred into cell culture plates containing Dulbecco’s Modified Eagle’s Medium (DMEM high glucose 4.5 g/L, HEPES modification), supplemented with 20% fetal calf serum (FCS), 4 mM L-glutamine, 50 µg/mL gentamicin, 100 U/mL penicillin, 100 µg/mL streptomycin, and 2.5 µg/mL amphotericin B (all from Sigma-Aldrich Chemie GmbH, Taufkirchen, Germany), and cultured in a humidified incubator at 37 °C, 5% CO_2_, and 95% air. After seven days, the bone chips were gently removed and the outgrown cells were cultured for an additional two weeks. Subsequently, the culture medium was removed and cells were rinsed twice with PBS. The cell culture plates were aspirated and stored at −80 °C until biochemical analysis. 

#### 2.5.4. Determination of the Number of Outgrown Cells via DNA Content

Frozen cell culture plates with outgrown cells were thawed for 20 min. on ice followed by lysis with 1% Triton X-100 in PBS for 50 min. on ice. Cell lysis was supported by sonication for 10 min. in an ice-cooled ultrasonic bath. 

The cell lysates were mixed with QuantiFluor^®^ dsDNA quantitation reagent (Promega, Madison, WI, USA) diluted 1: 800 in TE buffer (=10 mM TRIS and 1 mM EDTA) and incubated for 5 min. in the dark. The intensity of fluorescence was measured with the multifunction microplate reader (SpectraFluor Plus, Tecan, Crailsheim, Germany) at an excitation and emission wavelength of 485/535 nm. Relative fluorescence units were correlated with the cell number while using a cell calibration line to determine the number of outgrown cells. The determined cell number for the several drills was correlated to their experimentally produced bone chip mass as a related measurand for vitality.

#### 2.5.5. Data Expression and Statistics

All of the results are expressed as mean ± SEM. The number of drilling holes in each experiment is indicated by “n” and it is written in the caption of the diagrams for each investigated parameter. Vitality is expressed as the number of outgrown cells per mg bone chip mass. All the data were analysed while using a Kruskal–Wallis One Way Analysis of Variance on Ranks with a subsequent Student-Newman-Keuls or Dunn’s test while using SigmaPlot 12.3 (Systat Software, Erkrath, Germany). Differences were considered to be significant where *p* ≤ 0.05.

## 3. Results

### 3.1. Drill Geometry

The drill geometry of each tool of five commercial implant drill sets was characterized using optical profile projector devices (Table 1) and SEM. Table 2 summarizes the characterization of each drill. There were clear differences between the drills of different manufacturers regarding the drill shape, number of teeth, point, and helix angle. The cutting edge radii were comparable for all tools (<10 µm). All of the tools had a shank diameter of 2.35 mm and a dental tool clamping.

Particularly noteworthy was the different diameter distribution between the tools of the drill sets with a comparable final diameter. The set of manufacturer B only included three drills, whose diameters increased in very large increments. In contrast, the set of manufacturer D consisted of five drills whose diameters increased in smaller increments. The set of manufacturer C was considered in spite of its smaller final diameter due to their popularity on the market.

### 3.2. Influence of the Thrust Force

**Figure 4:**
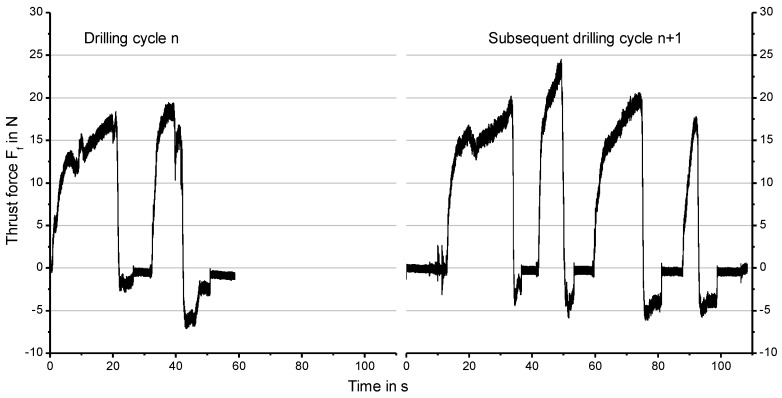
Exemplary signals of the thrust force *F_f_* of two subsequent drilling cycles for the same tool (Manufacturer B, Pilot drill).

Figure 4 shows the thrust force during manual drilling of two subsequent drilling cycles with identical conditions. The numbers of drilling cycles to obtain the drilling depths of 10 mm is recognizable. Even the operation times differ for the same tool. With the beginning of the drilling operation, the thrust force increases fast and is held approximately constant by the surgeon or rather the increase will be subsequently clearly lowered. The experience of the surgeon is visible in the form of the repetitive signal paths of the thrust force of the drilling operations. In addition, an abrupt decrease of thrust force by the surgeon to prevent the tool to get stuck is discernible in some drilling operations (Figure 4, right side).

**Figure 5:**
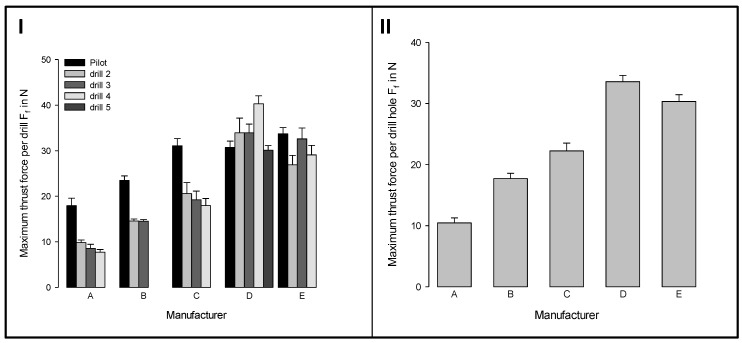
Maximum thrust forces (**I**) for different tools of five commercial drill sets and (**II**) for the whole sets; (mean ± SEM; n = 8).

The signals of the thrust force of the different tools of the five commercial drill sets were analyzed with regard to their maximum values (Figure 5). The set of manufacturer A generated the lowest thrust forces of all. It is remarkable that drill Set B showed low maximum forces, despite large increments of the diameter of the drills. The highest forces were measured for sets D and E.

### 3.3. Influence of the Temperature

**Figure 6:**
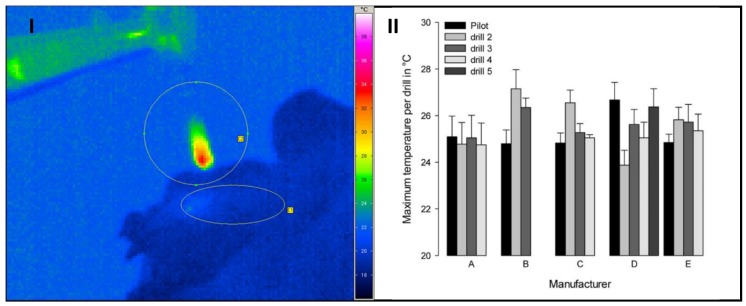
Thermogram after drilling with temperature distribution at the tip of the drill (circle) and at the entrance of the drilled hole (ellipse) (**I**). Maximum temperature with the different tools of five commercial drill sets measured at the tip of the drill (**II**) (mean ± SEM; n = 4).

The maximum temperatures were measured at the drill tip when the tool emerged from the hole after the drilling process. These temperatures were below 28 °C in any case (Figure 6), so that tissue damaging influences on the extracted chip material can be excluded due to the selected mechanical-technological parameters. No significant differences between the sets were observed. The challenge here is that the emissivity changes in different ways due to the moistening of the drills with fluids or residues of particle and does not remain constant.

### 3.4. Theoretical and Experimental Bone Chip Mass

**Figure 7:**
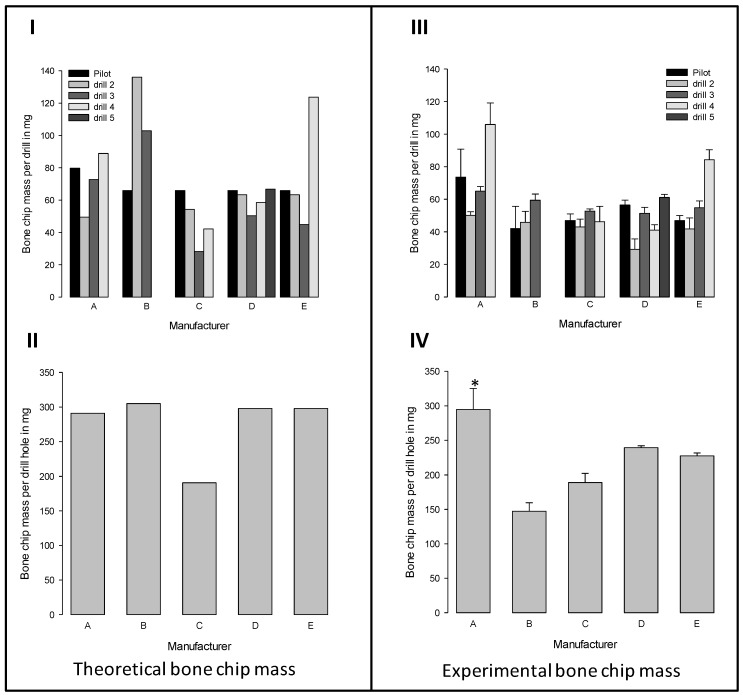
Comparison of (**I**, **II**) theoretically expected and (**III**, **IV**) experimentally produced bone chip mass. The upper row presents the results for each drilling tool of the five commercial implant drill sets (**I**, **III**) The lower row shows the total bone chip masses produced with a whole drill set (**II**, **IV**). (mean ± SEM; n = 3). * significant difference compared to all other drill sets (Kruskal-Wallis One Way Analysis of Variance on Ranks with subsequent Student-Newman-Keuls test, *p* ≤ 0.05).

In Figure 7**I** the theoretical bone chip masses for each drilling tool of five commercial drill sets are shown. The theoretical masses vary according to the different tool diameters since drilling depth and tissue density remain the same for all tools.

The theoretical mass of each tool is mainly dependent from the diameter increments of the tools within the set. The drills within the set from manufacturer D all produced approximately the same theoretical bone chip masses due to almost uniform diameter increments and the need of five tools to achieve the final diameter of the drilling hole. In contrast, the three tools from manufacturer B created relatively high theoretical bone chip masses due to the larger diameter increments. By summing up the masses of the individual tools, the total theoretical mass of bone chips was obtained, which might be produced with a whole drill set (Figure 7**II**).

Despite the different diameter distribution between the tools within the drill sets, all sets, except Set C, produce the same total theoretical mass, as these sets have a comparable final diameter (Figure 7**II**). The experimental verification of the actually produced bone chip masses (Figure 7**III,IV**) showed differences between the individual tools of the five commercial implant drill sets, as were expected from the calculation of the theoretical masses (Figure 7**II**).

Furthermore, we found differences between the theoretical and experimental bone chip masses. For most tools, the experimentally obtained values were below the theoretically possible masses (Figure 7**I** versus **III**). These differences become particularly clear when the masses of the individual drills in a set were summed up (Figure 7**II** versus **IV**). For the sets B, D, and E, the experimentally obtained masses were lower than the theoretical bone chip masses, with the highest difference for set B. Remarkable was that the set of manufacturer B produces half the bone mass of set A in the experiments, although set B theoretically produces more bone chip mass than set A. In contrast, for set A and C, the experimentally obtained masses corresponded to the theoretical masses (Figure 7**II** versus **IV**).

### 3.5. Bone Chip Size and Their Geometry

**Figure 8:**
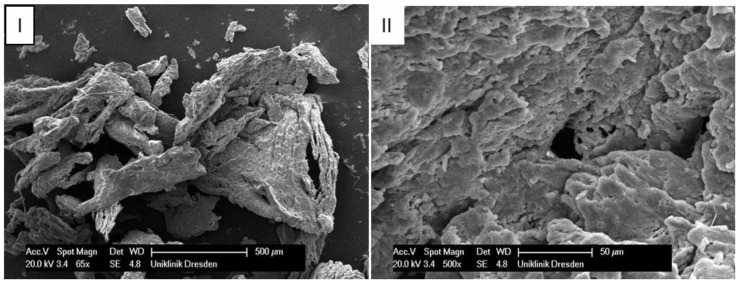
Scanning electron microscope images of bone chips in wet room mode (**I**) and sputtered with gold in high vacuum (**II**).

The size of the bone chips produced varied widely. Particles ranging in size from micrometres to millimetres were present beside each other (Figure 8**I**). A classification of the chips with regard to shape and size was not possible due to the sticking of the bone chips (Figure 8**II**). A non-destructive separation of the chips before application to the sample carrier was not possible mechanically or by ultrasonic cleaning with “SONOREX" (Bandelin, Berlin, Germany).

### 3.6. Vitality of the Bone Chips

**Figure 9:**
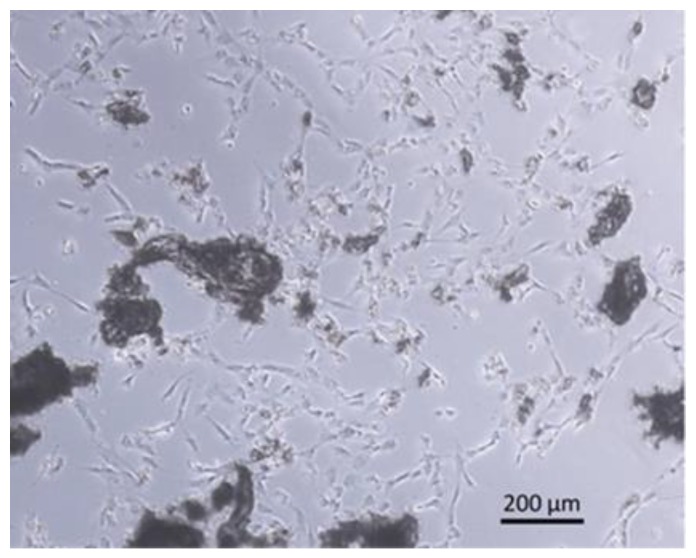
Scanning electron microscope image of cells grown out of in vitro cultured bone chips.

To determine the vitality of the collected bone chips, we transferred the bone chips into cell culture vessels and cultivated them in vitro. After one week, an outgrowth of cells from bone chips was observed (Figure 9).

**Figure 10:**
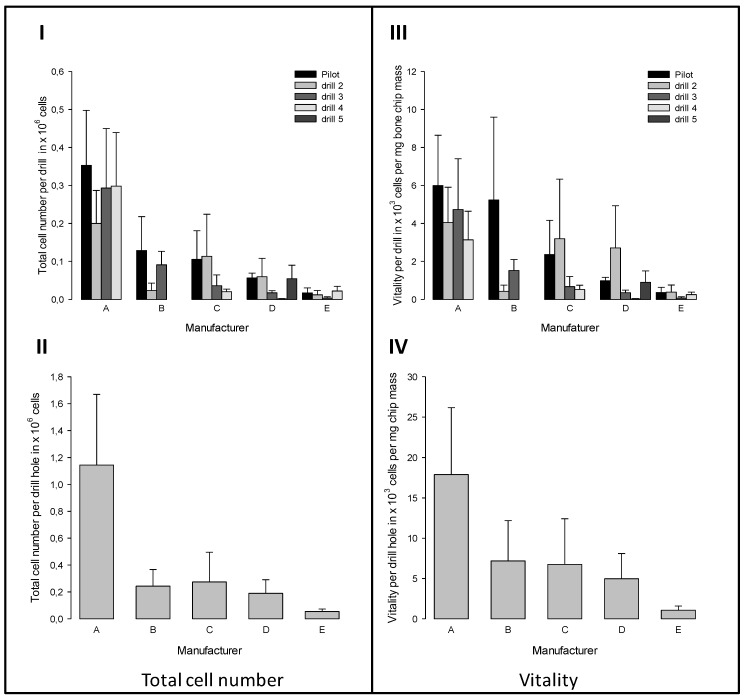
Total numbers of (**I**, **II**) outgrown cells and (**III**, **IV**) vitality of collected bone chips (displayed as number of outgrown cells per mg drilled bone chip mass). The upper row presents the results for each drilling tool of the five commercial implant drill sets (**I**, **III**). The lower row shows the sums for the respective drill sets (**II**, **IV**). (mean ± SEM; n = 3).

After four weeks, the number of outgrown cells was determined. Like the experimentally produced bone chip masses, the numbers of outgrown cells also varied considerably (Figure 10**I**,**II**). Obviously, most cells grew out of the chips that were generated with the individual drills of the set A (Figure 10**I**). In contrast, the lowest number of cells grew out from chips that were produced by tools of set E. Again, these differences are more clear from the summation of the individual values (Figure 10**II**). By correlating the total cell numbers (Figure 10**I**) with the experimentally determined bone chip masses (Figure 7**III**), a related measurand of the vitality of the bone chips is obtained. The drills of set A generate chips with the greatest vitality and drills of the set E, chips with very low vitality (Figure 10**IV**).

## 4. Discussion

The present paper focuses on the investigation of the effect of drill geometry and resulting loads of five commercial implant drill sets on mass and vitality of bone chips produced during the preparation of the implant site to reuse them as a highly vital, autologous augmentation material. For this purpose, we first characterized the various drilling tools to correlate their geometrical properties, suitable process parameters during and after drilling subsequently with the related measurand for the vitality of the chips produced.

Bone chips that are suitable for augmentation should be as vital as possible. The number of outgrown cells was correlated with the weight of the bone chips seeded in order to obtain a related measurand for vitality of the bone chips harvested. As a result, differences in vitality must be due to other factors than to the different bone chip masses that are produced by the various drills.

Bone chips that are produced with the drill set from manufacturer A showed the highest vitality. More than twice as many cells per mg of chip mass grew out than from bone chips generated with sets from other manufacturers.

A hypothesis of this work was the assumption that, with the same final diameter of the last drills of the respective sets, same masses of bone chips are produced and this effect is not dependent on the drill set configuration or tool geometry. We first calculated the theoretically producible chip mass based on the diameter measurement of the individual drills in order to verify this assumption. The next step was the experimental verification of the theoretically expected chip masses by conducting hand-guided drillings on the lower jaws of pigs with the drill sets and weighing the bone chips.

The experimental verification revealed similarities and differences between the theoretically expected and actually produced bone chip masses. While for the sets A and C, the actually produced masses corresponded with the theoretically expectable masses, the experimentally obtained values for set B, D, and E were below the theoretically possible masses. This means that less bone material was cut off by these drill sets, or, ablated bone material was not extracted from the drilling hole and weighed. The former might be due to unavoidable inaccuracies in the manual drilling process.

A further characteristic of the hand-guided drilling process is that the feed motion does not run exactly along a straight line. Thereby, the tool geometry has significant influence on the guidance of the tool during drilling, since it can promote or prevent the tilting of the drilling axis. A drift away from the ideal drilling axis and thus a deviation from the theoretically assumed shape of the drilling hole would increase the expected chip mass. The present results do not seem to support such a tool geometry-related drifting effect. Rather, the question remains as to where the produced bone chips have gone. It can be assumed that not all of the chip material was collected from the drilling hole. Due to the special structure of cancellous bone, it is possible that ablated bone chip material was compressed into the medullary cavities/ marrow spaces between the bone trabeculae or into fractures that are caused by the drilling process, and thus cannot be extracted. However, this phenomenon also relies on tool geometry. The removal of the bone chips is achieved via the flute of the drill, the design and composition of which is an essential part of the tool geometry. This means: insufficient chip transport due to unfavourable tool geometry causes compressing into the surrounding tissue.

We assume that differences in the vitality as well as the bone chip masses are due to the different tool geometries and set configurations. The sets varied in different geometry parameters (influencing variables, see Table 2). Despite comparable final diameters of the last drills (except Set C), these drill sets differ regarding set configuration, diameter increment, and tool geometry (geometry parameters/influencing variables, see Table 2). It should be noted that the geometries of the pilot drills and the subsequent drilling tools often differ in more than one parameter.

Various studies by other authors indirectly point to the relationship between geometry and chip size. Lim et al. (2017) and Park et al. (2010) were able to prove, on bovine rib bones, that the chip size depends on the implant drill design [18,24]. Furthermore, some studies investigated the relationship between chip size and osteogenic and osteoconductive potential of the bone chips [14,21,25,26]. Bone particles that range in size from 100 to 500 µm have an osteoconductive rather than an osteogenic effect [19,25,27]. However, studies on rabbits show that particle size in the range of 800–2000 µm is more suitable for bone formation than smaller particle sizes [19,27]. Hence, it seems that, the bigger the particles, the more osteogenic they are [28]. However, it can be assumed that a mixture of large and small bone particles combines the respective advantages of the different particle sizes: the osteoconductivity of the small particles with the osteogenicity and mechanical stability of the large particles. Thus, the tool geometry appears to correlate indirectly with vitality by influencing the particle size of the bone chips obtained.

The chip size and also their geometry are dependent both on the tool geometry and on the process parameters. The assessment base is the uncut chip thickness *h* (Equation (5)), which is geometrically determined by the point angle *σ* and technologically by the feed per tooth *f_z_*.

(5) $$h=f_{z}\cdot sin\frac{\sigma }{2}$$

The uncut chip thickness acts as a theoretical value and it is linked to the real measurable chip thickness via the chip compression factor. The amount of this unit of length must exceed a minimum value that is specified by the size of the bone cells. Otherwise, the bone cells will be disrupted and the vitality will be reduced.

In addition to the geometry of the tool, the drilling process itself plays a role. The feed per tooth *f_z_* defines the achievable chip thickness (Equation (6)). The feed per tooth varies since this is a manual drilling process, as does the chip thickness. Due to the multiple changes in geometry parameters between the tools and the unconstant feed per tooth and, subsequently, the feed rate, the effect sizes cannot be clearly separated from each other. However, a qualitative difference of the produced chip consistencies between the tools was noticeable in the course of the test.

Therefore, we also wanted to determine the particle size distribution of the bone chips that were produced in this study. The determination of the particle size proved to be difficult and it could not be reliably carried out. This was partly due to the extreme size variation of the particles (30 µm to 5 mm) and partly to the small volume of the chip material obtained. In fact, there is currently no suitable method available for accurately quantifying the entire range of particle sizes. In addition, this would require chip masses or volumes that are practically impossible to obtain. Various bone chip consistencies (depending on the tool used) were observed. While some tools produced a loose powder, others showed a strong compression of the bone chips. This manifested itself as strands that adhered in the flute. Possibly, this compression could be a cause for the damage of the containing bone cells, which would subsequently lead to reduced vitality.

There is evidence of a correlation between vitality and thrust force. The geometry and the material-side design of the tool have crucial influence on the operation-specific suitability in general and with regard to the production of suitable augmentation material in particular.

The extended Kienzle function is used to calculate the thrust force *F_f_*. This function reflects the functional relationship between the geometric design of the tool and the resulting thrust force (Equation (6)).

(6) $$F_{f}∼\;K_{1f}\cdot {\left(f_{z}\cdot sin\frac{\sigma }{2}\right)}^{K_{2f}}$$

The values of the constants *K_1f_* and *K_2f_* of the Kienzle function are tool and material dependent. The geometric design of the flute, the design of the secondary cutting edges, and the tribological behavior between the drilling tool and the bone largely determine them. Geometric parameters cannot be one-dimensionally displayed on the tool. In addition, the design of drilling tools is implemented via undocumented free-form surfaces while using multiple grinding processes. Therefore the correlations are very complex and cannot be broken down to a single parameter.

However, it was clearly shown that the pilot drill (first drill during preparation of the implant site) is the most important in terms of thrust force. The reason for this is that, in contrast to the following drilling cycles, it is a drilling into the solid material.

The thrust forces are up to 80% higher when compared to other tools of the respective set (see Figure 5**I**—Set A). This is due to the contact of the chisel edge in the area of the rotation axis, where the cutting speed is zero and the flute has a small cross-section due to the geometric dimensions. Furthermore, despite comparable tool diameters, the thrust forces considerably differ between two sets. For example, all of the measured forces of the drilling sets B to E are at least 30% higher than the values of Set A.

The process measurands are used as indirect parameters to assess the performance of a drill set since the geometric and tribological description is too complex and individual tool-specific parameters, which have a direct influence on the vitality, cannot be determined. In fact, there is a statistically proven correlation between thrust force and vitality. This correlation was also proven by Sugita et al. (2019) for bovine cortical bone [29].

Although an expectation was given, it was not possible to demonstrate a correlation between tool geometry, temperature development, and vitality of the bone chips. As became evident, IR thermography is only suitable to a limited extent for the thermal examination of the manual drilling processes and diameter ranges analyzed here.

The increase in temperature that is caused by friction during the drilling process can influence the bone tissue and the bone chips extracted. We worked with low speeds in our setup in order to keep the possible damages as low as possible. Nevertheless, one objective of this work was to investigate the temperature development during the drilling process. However, we measured no significant temperature differences. There are several reasons for this:

1. The material of the drilling tools has a high thermal conductivity. In addition, the drills are removed relatively slowly from the drilling hole. This means that the withdrawal speed is low. The position of the drilling tools varies in the area of the focal point of the IR camera due to the manual drilling process. Only minor temperature changes are measured in the area of the tool cutting edge due to the interaction of these factors.
2. The measured temperature is influenced, among other things, by the emission coefficient, which is effective in the cutting area. This, in turn, varies depending on the sample, since the cutting area is generally wetted with bone material, drill chips, tissue, or blood.
3. The effect size is too small to statistically confirm the differences. In the opinion of some users/surgeons, water-cooling of the drill bit should be dispensed and, in return, the rotational speed should be lowered to obtain bone chips that are as vital as possible for augmentation.

It seems that the lower cutting speeds that were produced in this way already lead to a considerable reduction in frictional heat, so that hardly any temperature differences can be detected.

## 5. Conclusions

Various factors influence the outgrowth of bone cells from the bone chips and thus the vitality. We have investigated the factors tool and chip geometry as well as mechanical and thermal loads to characterize the factors influencing the vitality of the bone chips obtained during drilling under practical conditions.

It could be proven that there is a correlation between the related measurand vitality and thrust force. The thrust force is influenced by the cutting behavior of the tool, which in turn depends on the geometry of the tool. The tool geometry has an influence on the vitality of the bone chips obtained due to this relationship.

The tool geometry and configuration of Set A appears to be superior to the other drill sets that were investigated in terms of the vitality of the bone chips harvested and thus their suitability as augmentation material. This set actually produced the largest chip mass of all sets, which also corresponds to the theoretically predicted chip mass. Obviously, the tool geometry and configuration of this drill set cause a gentle ablation of the bone tissue with reduced damage character due to highly increased temperatures or compressions of the surrounding tissue and thus leading to a superior vitality of the bone chips.

The influence and effect size of other factors, like tool geometry with its specific parameters, chip geometry, and the measurement of the local temperature at the drill tip, need to be further investigated. A better understanding of those influence factors could lead to damage minimized drilling of bone tissue, which, for example, could positively affect the anchorage of bone screws and with that the primary stability of osteosynthesis systems [30]. Nevertheless, the used method was appropriate to verify a tool set with the aim of producing vital bone chips for the practical use.

## Figures and Tables

**Table 1:** Technical specifications of the optical profile projectors used to characterize the drill geometry.

Attribute	Device 1	Device 2
Manufacturer	Mitutoyo GmbH (Japan)	Schneider Messtechnik GmbH (Bad Kreuznach, Germany)
Model	PJ 300	ST 300
Resolution	1 µm	0.5 µm
Magnification	10×, 20×, 50×, 100×	10×, 20×, 50×, 100×

**Table 2:** Characterization of the drill geometry of five different drill sets (A–E).

**Set**	**Drill Shape**	**Tool Diameter in mm**	**Number of Teeth z**	**Point Angle σ in °**	**Helix Angle λ_s_ in °**	**Flute Length in mm**	**Shape of the Drill Tip**
**Set A**	twist	2.2	2	122	25	14	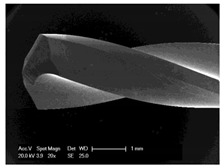
	twist	2.8	3	122	25	14	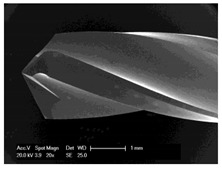
	twist	3.5	3	122	25	14	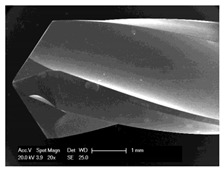
	twist	4.2	3	130	25	14	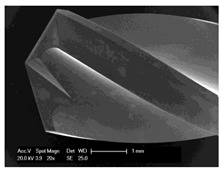
**Set**	**Drill Shape**	**Tool Diameter in mm**	**Number of Teeth z**	**Point Angle σ in °**	**Helix Angle λ_s_ in °**	**Flute Length in mm**	**Shape of the Drill Tip**
**Set B**	twist	2	2	90	20	16	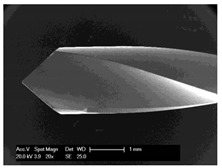
	tapered	3.5	4	120	0 (straight)	10	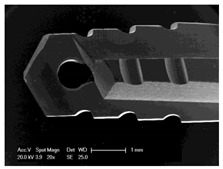
	tapered	4.3	4	120	0 (straight)	11	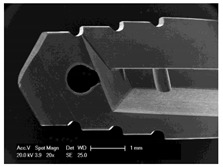
**Set**	**Drill Shape**	**Tool Diameter in mm**	**Number of Teeth z**	**Point Angle σ in °**	**Helix Angle λ_s_ in °**	**Flute Length in mm**	**Shape of the Drill Tip**
**Set C**	twist	2.0	2	118	15	14	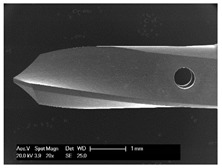
	conical twist	2.7 (3.0)	2	125, 40 (segmented)	15	14	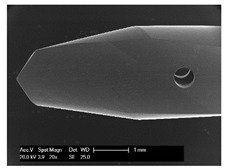
	conical twist	3.0 (3.4)	2	125, 40 (segmented)	15	14	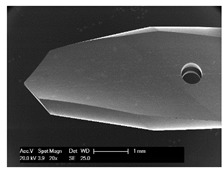
	conical twist	3.4 (3.8)	2	125, 40 (segmented)	15	14	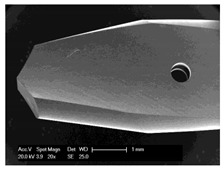
**Set**	**Drill Shape**	**Tool Diameter in mm**	**Number of Teeth z**	**Point Angle σ in °**	**Helix Angle λ_s_ in °**	**Flute Length in mm**	**Shape of the Drill Tip**
**Set D**	conical tapered	1.7–2.8	3	115, 9	0 (straight)	14	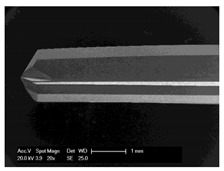
	twist	2.0	2	118	20	14	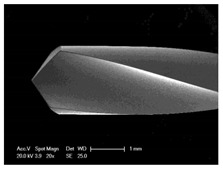
	tapered	3.3	4	140, 15	0 (straight)	11	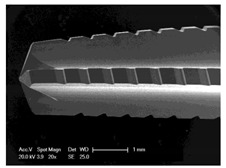
	tapered	3.8	4	140, 6	0 (straight)	16	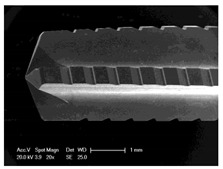
	tapered	4.3	4	140, 6	0 (straight)	16	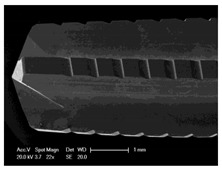
**Set**	**Drill Shape**	**Tool Diameter in mm**	**Number of Teeth z**	**Point Angle σ in °**	**Helix Angle λ_s_ in °**	**Flute Length in mm**	**Shape of the Drill Tip**
**Set E**	twist	2	2	103	25	15	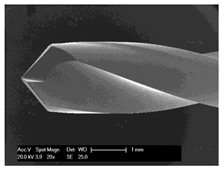
	stepped, twist	2.8	3	118	25	15	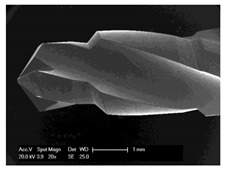
	twist	3.25	3	140, 90 (segmented)	20	16	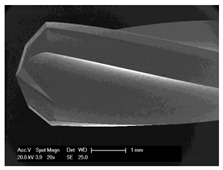
	twist	4.25	3	140, 90 (segmented)	20	16